# Survival With Lenvatinib for the Treatment of Progressive Anaplastic Thyroid Cancer: A Single-Center, Retrospective Analysis

**DOI:** 10.3389/fendo.2020.00599

**Published:** 2020-09-02

**Authors:** Soo Young Kim, Seok-Mo Kim, Jun Won Kim, Ik Jae Lee, Tae Joo Jeon, Hojin Chang, Bup-Woo Kim, Yong Sang Lee, Hang-Seok Chang, Cheong Soo Park

**Affiliations:** ^1^Department of Surgery, Thyroid Cancer Center, Gangnam Severance Hospital, Yonsei University College of Medicine, Seoul, South Korea; ^2^Department of Radiation and Oncology, Thyroid Cancer Center, Gangnam Severance Hospital, Yonsei University College of Medicine, Seoul, South Korea; ^3^Department of Nuclear Medicine, Thyroid Cancer Center, Gangnam Severance Hospital, Yonsei University College of Medicine, Seoul, South Korea

**Keywords:** lenvatinib, thyroid carcinoma, anaplastic thyroid cancer, retrospective study, tyrosine kinase inhibitor

## Abstract

**Background:** Survival rates for anaplastic thyroid cancer (ATC) have not improved in the past four decades; however, preliminary clinical data indicate that lenvatinib may provide efficacy benefits for patients with ATC. This real-world study aimed to define the potential role of lenvatinib in ATC by examining the impact of treatment administered alongside existing therapies.

**Methods:** This was a retrospective, single-center analysis of Korean patients with confirmed ATC who received lenvatinib between October 2015 and February 2018. Eighteen patients were included (mean ± standard deviation age, 64.9 ± 11.1 years; 61.1% female). Six [33.3%] had resectable disease that progressed after a combination of surgery, radiotherapy, and chemotherapy, and 12 [66.7%] had unresectable disease that progressed after radiation treatment and chemotherapy. Study endpoints were overall survival (OS) and change in volume of the largest tumor assessed via imaging.

**Results:** Median OS for the 18 lenvatinib-treated patients was 230 days (range 64–839 days). Survival rates at 6 months and 1 year were 61.1 and 22.2%, respectively. Three patients (16.7%) survived beyond 1 year; 15 patients died, of whom four (26.7%) had local disease and 11 (73.3%) had distant metastasis. Two patients (11.1%) had tumor volume increases of 9–10%. The other 16 patients (88.9%) had tumor volume reductions of 2–69%. Six patients (33.3%) had tumor volume reductions ≥50%.

**Conclusions:** In patients with ATC who had progressed on prior therapy, addition of lenvatinib could improve survival duration and reduce tumor volume. Further studies of lenvatinib in ATC are warranted.

## Introduction

Anaplastic thyroid cancer (ATC) is a rare, highly aggressive malignancy composed of undifferentiated thyroid follicular cells (1). It is a devastating disease that carries a very poor prognosis (2). ATC accounts for ~2% of all thyroid neoplasms, but is responsible for up to 50% of thyroid cancer mortality (3). In addition to local invasion, ATC often metastasizes to regional lymph nodes and distant sites (4).

A recent analysis of the Surveillance, Epidemiology, and End Results (SEER) database suggested that the incidence of ATC may be increasing (5), which is likely caused by increased detection of tumors due to improved diagnosis guidelines. There were 1,527 patients diagnosed with ATC between 1973 and 2014; in 1973 the age-adjusted incidence rate was 0.2 per 1,000,000 people, compared with a rate of 1.2 per 1,000,000 in 2014. This corresponds to an average annual percentage increase of 3.0% (5). Median survival with ATC is reported to be around 5 months, with fewer than 20% of patients surviving for more than 1 year (6). Survival rates have not improved in the past four decades despite considerable research and the availability of new treatments (5). Analysis of SEER data indicated that the median disease-specific survival time was 4 months during the period of 2010–2014, which was almost unchanged from the median 3-month survival time during the period of 1975–1979 (5).

Current treatment guidelines for ATC suggest that therapy should include surgery for resectable disease, locoregional radiotherapy, and systemic chemotherapy (adjuvant or second-line) (7, 8). Targeted agents such as the B-Raf inhibitor dabrafenib, the mitogen-activated protein kinase (MAPK) inhibitor trametinib, and the tropomyosin receptor kinase (TRK) inhibitor larotrectinib may be useful in patients with specific genetic mutations (7). The multitargeted tyrosine kinase inhibitor (TKI) lenvatinib is currently recommended for use in patients without curative options who have no response to other agents (7).

Lenvatinib was approved as a monotherapy for the treatment of differentiated thyroid cancer (2015), in combination with everolimus for the treatment of advanced renal cell carcinoma (2016) and most recently as a first-line treatment for unresectable hepatocellular carcinoma (2019) (9–12). Preliminary clinical data also suggest that lenvatinib may provide efficacy benefits for patients with ATC (10, 13, 14). In a phase 2, single-arm, open-label study in 17 patients with ATC, the median progression-free survival was 7.4 months, the median overall survival (OS) was 10.6 months, and the objective response rate was 24%. Although all patients reported treatment-emergent adverse events (AEs), the majority were manageable with dose adjustments (10, 13, 14). As of yet, however, there is no established protocol for the treatment of patients with ATC, and lenvatinib's place in the treatment algorithm is unclear.

This retrospective, real-world analysis was conducted to examine the impact of lenvatinib when administered alongside existing local and systemic treatments to further define the potential role of this agent in improving outcomes for patients who have few other treatment options.

## Materials and Methods

### Study Design

This was a retrospective, single-center analysis of patients with confirmed ATC (diagnosed between January 2008 and December 2017) who received lenvatinib treatment at the Gangnam Severance Hospital (Seoul, South Korea) between October 2015 and February 2018. For eligible patients, electronic medical records were reviewed to extract data on clinical characteristics, including age, tumor characteristics, prior treatment, and treatment outcomes.

All procedures involving patients were performed in accordance with the ethical standards of institutional regulations and all applicable local/national regulations, and with the 1964 Helsinki declaration and its later amendments or comparable ethical standards. In accordance with the Bioethics and Safety Act of Korea, formal consent was not required for this type of retrospective, observational analysis.

### Patients

Patients with confirmed ATC who received treatment with lenvatinib after progression with previous anticancer therapy were eligible for inclusion in the analysis. Pathologic confirmation of ATC was required to confirm the diagnosis of ATC, either through surgery or via open biopsy or core needle biopsy. Patients who had received treatment with sorafenib were excluded. No additional exclusion criteria were applied. Patients were followed up for at least 1 year or until death occurred.

### Treatment

**Figure 1:**
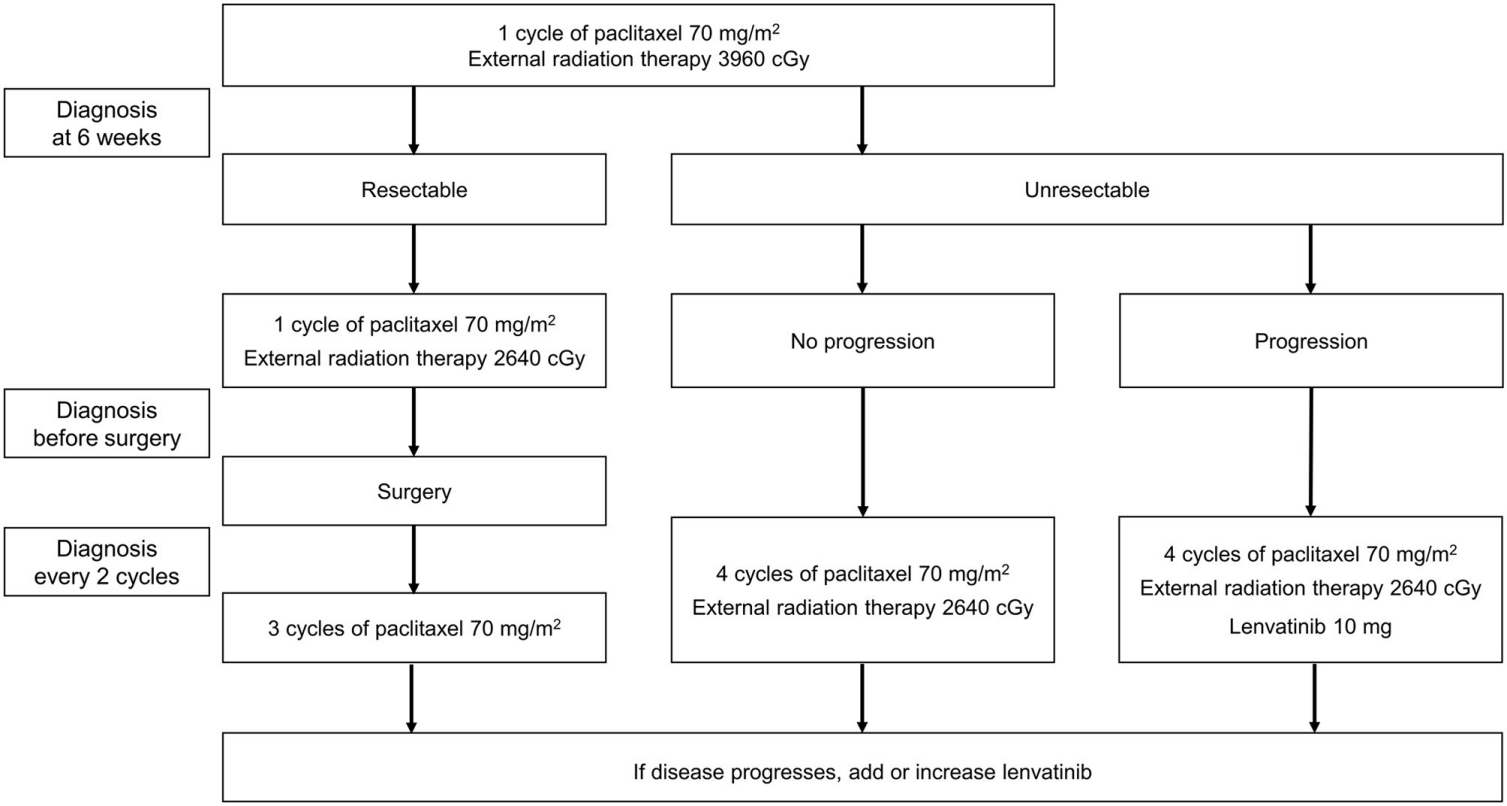
Anaplastic thyroid cancer treatment protocol followed at the Gangnam Severance Hospital.

Lenvatinib was added to each patient's existing treatment. The treatment protocol followed in our hospital for patients with ATC is shown in Figure 1. Administration of symptomatic therapy was allowed for patients who experienced treatment side effects.

Surgery was planned for patients in whom the primary tumor was resectable and there was no distant metastasis. The patient was administered one cycle of paclitaxel (one injection of 70 mg/m^2^ each week for 3 weeks followed by a break of 1 week, with concomitant intensity-modulated radiation therapy [IMRT] at a dose of 3,960 cGy). After 6 weeks, tumor imaging (using positron emission tomography [PET], computed tomography [CT], or magnetic resonance imaging [MRI]) was used to determine whether surgery was appropriate. One further cycle of paclitaxel (70 mg/m^2^) plus IMRT (2,640 cGy) was administered prior to surgery, and three cycles of paclitaxel (without IMRT) were administered post-surgery. Routine diagnosis was conducted every two cycles, and if progression was observed, lenvatinib 10 mg/day was added to the treatment regimen. The maximum number of post-surgical paclitaxel cycles was six per patient.

In patients who were judged to have unresectable disease after the first 6 weeks, paclitaxel chemotherapy and IMRT were continued, with routine imaging performed every two cycles. If progression occurred, lenvatinib 10 mg/day was added. If progression continued, the dose of lenvatinib could be increased to 20 mg/day, and finally to 24 mg/day. The starting dose of lenvatinib was 10 mg/day, as clinical experience has shown that numerous adverse events, including leukopenia and generalized muscle weakness, were reported when starting with a maximum dose of lenvatinib in a combination therapy.

### Efficacy Outcomes

The primary study endpoint was OS, defined as the duration between the start of lenvatinib treatment until death from any cause. Survival rates at 6 months and 1 year were also calculated. The secondary endpoint was change in volume of the largest tumor (primary or metastasis) assessed using PET, CT, or MRI.

### Statistical Methods

As far as possible, all patients who received lenvatinib treatment in our institute between October 2015 and February 2018 were enrolled in the study; the only exclusions were for prior treatment with sorafenib. No sample size estimations or power calculations were performed.

For the analysis of study data, categorical variables were described by frequency and proportion; summary statistics (median, range) were used to report continuous data. Survival curves were generated using the Kaplan–Meier method based on the log-rank test. As this was a retrospective analysis, no formal statistical comparisons were performed.

## Results

### Patients

**Figure 2:**
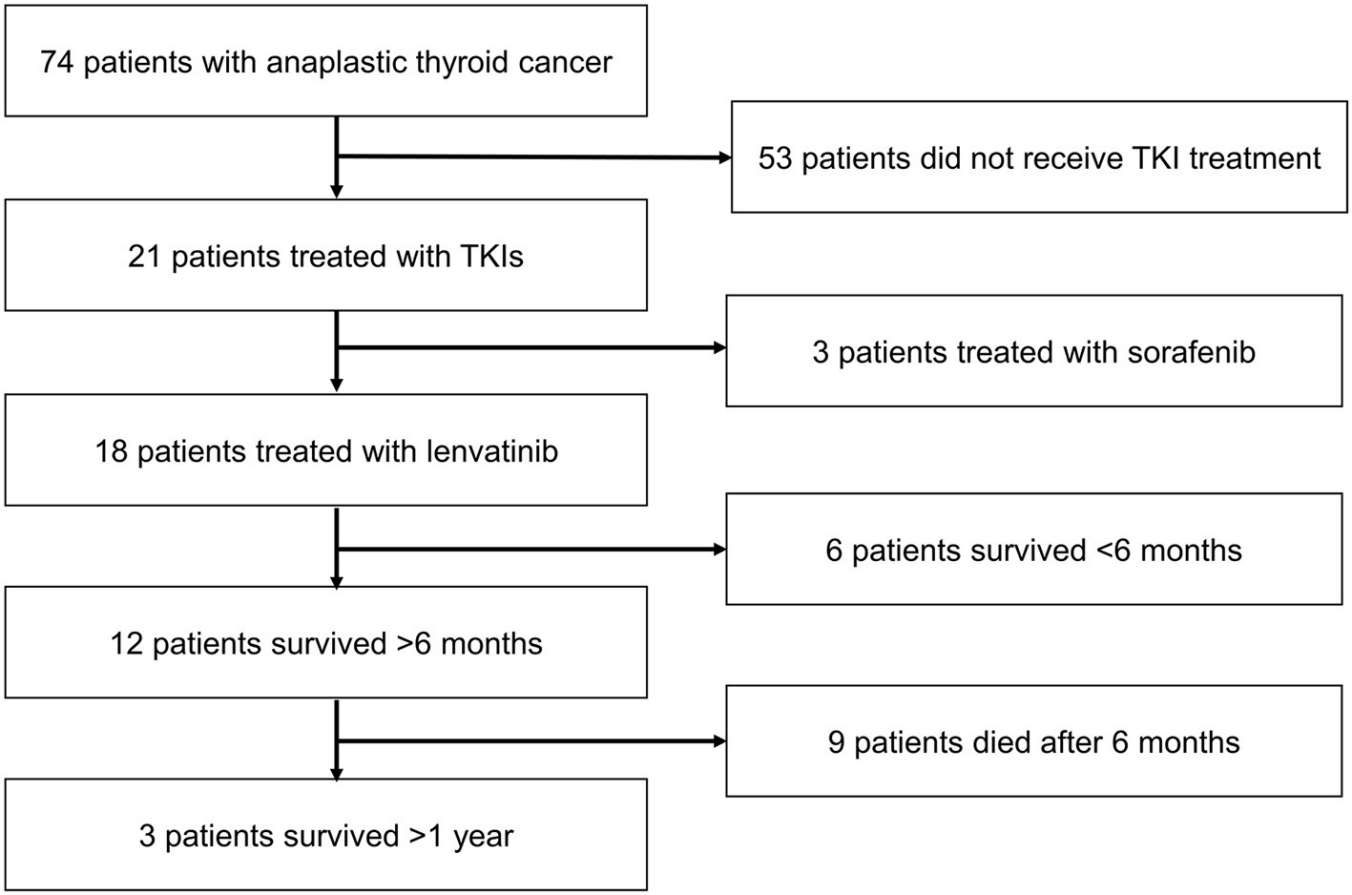
Patient disposition. TKI, tyrosine kinase inhibitor.

The patient disposition is shown in Figure 2. A total of 74 patients with ATC were evaluated, of whom 53 were not treated with a TKI. Of the 21 patients who received TKI treatment, three received sorafenib; 18 patients received lenvatinib and formed the analysis population.

Patient demographics and disease characteristics are described in Table 1. The mean age was 64.9 years, 61.1% of patients were female, the majority of patients (77.8%) had an Eastern Cooperative Oncology Group performance score (ECOG PS) of 0, and 94.5% had stage IVC ATC at diagnosis.

**Table 1:** Patient characteristics and clinical features.

**Variable**	**Patients *N* = 18**
Age (years), mean ± SD (range)	64.9 ± 11.1 (42–86)
Male: female ratio, *n* (%)	7 (38.9):11 (61.1)
ECOG PS, *n* (%)	
0	14 (77.8)
1	3 (16.7)
2	1 (95.5)
Cancer stage at diagnosis, *n* (%)	
IVA	0
IVB	1 (5.5)
IVC	17 (94.5)
Tumor size (cm), mean ± SD	
Baseline	5.2 ± 1.6
Post-therapy	3.5 ± 2.0
Combined therapy, *n* (%)	
Surgery	6 (33.3)
Chemotherapy	18 (100)
External radiation	18 (100)
Survival time (days), median (range)	230 (64–839)
Survival rate, *n* (%)	
6 months	11 (61.1)
1 year	4 (22.2)
>1 year	3 (16.7)
Patients who died, *n* (%)	15 (83.3)
Local disease	4 (26.7)
Distant metastasis	11 (73.3)

*ECOG PS, Eastern Cooperative Oncology Group performance score; SD, standard deviation*.

Lenvatinib was administered to patients with resectable disease (*n* = 6 [33.3%]) that had progressed after a combination of surgery, radiotherapy, and chemotherapy, and to patients with unresectable disease (*n* = 12 [66.7%]) that had progressed after radiation treatment and chemotherapy. All patients (100%) had received radiation therapy and paclitaxel prior to starting lenvatinib. Detailed treatment data for individual patients are provided in Supplementary Material Table S1. The treatment timeline is shown in Supplementary Material Table S2.

### Primary Outcome

**Figure 3:**
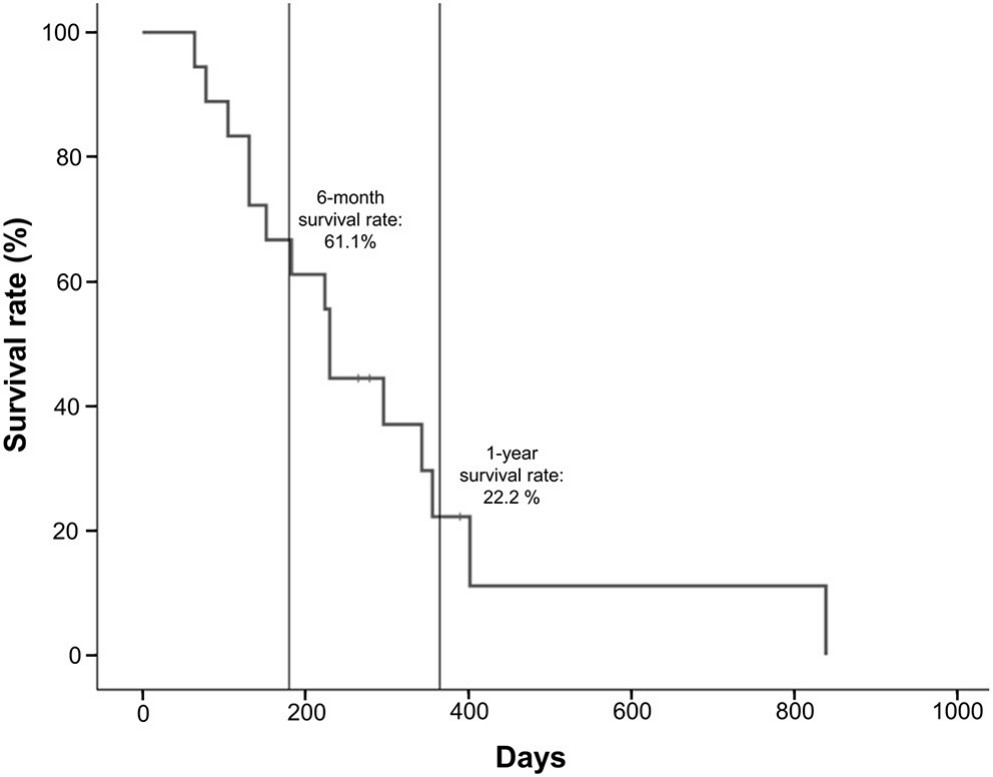
Survival rates of anaplastic thyroid cancer patients treated with lenvatinib.

The median OS for the 18 patients who received lenvatinib was 230 days (range 64–839 days) (Table 1). Survival rates were 61.1% at 6 months and 22.2% at 1 year (Figure 3). Three patients (16.7%) survived beyond 1 year with lenvatinib treatment. Of the 15 patients who died, four (26.7%) had local disease and 11 (73.3%) had distant metastasis.

### Secondary Outcome

**Figure 4:**
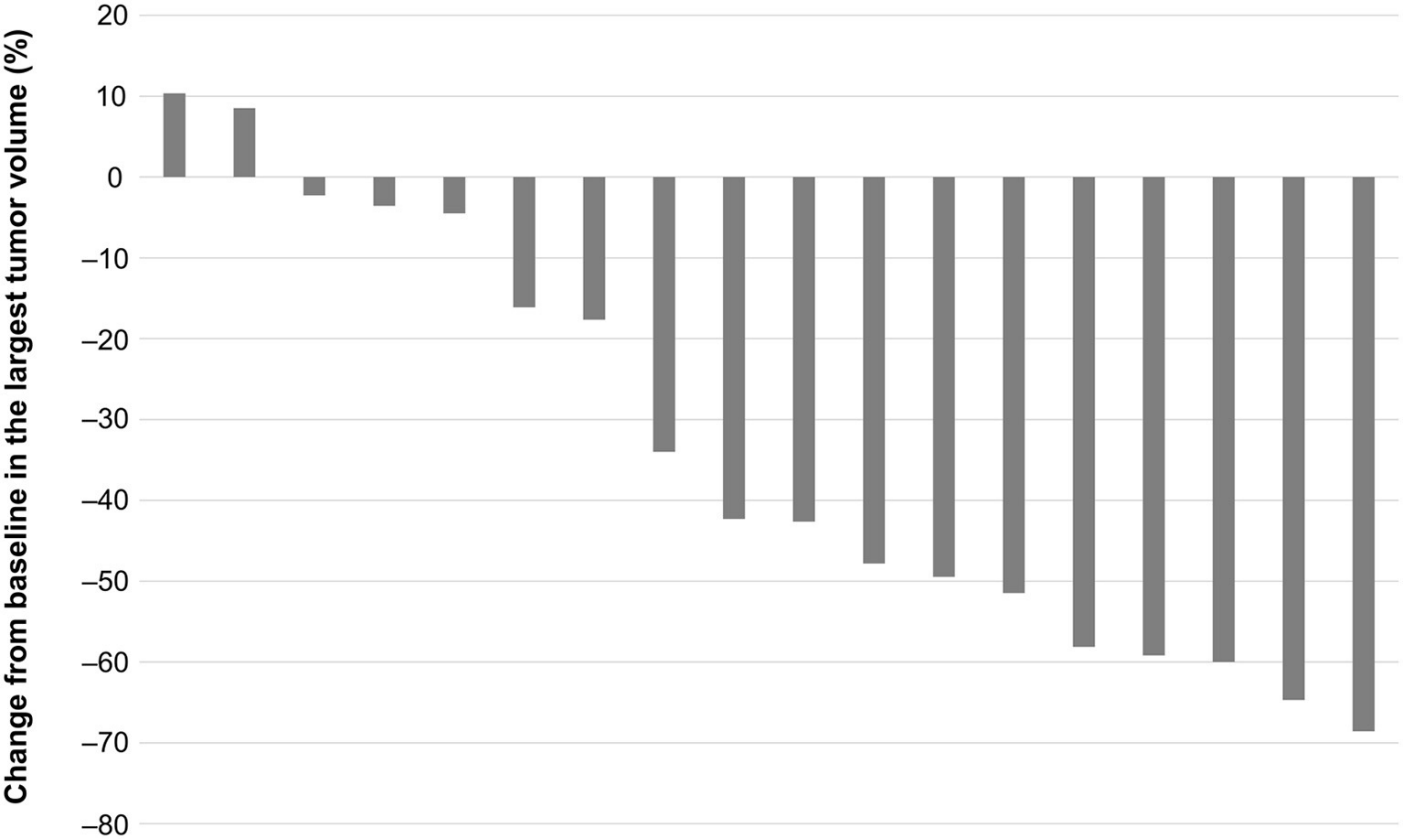
Change in volume of largest primary or metastatic tumor from the start to the end of lenvatinib treatment.

The percent change from baseline in the volume of the largest tumor (metastasis or primary) during lenvatinib treatment is shown in Figure 4. Two patients (11.1%) had increases in tumor volume of 9–10%. The other 16 patients (88.9%) had reductions in tumor volume from 2 to 69%. Six patients (33.3%) had tumor volume reductions ≥50%.

## Discussion

This retrospective analysis evaluated the role of the TKI lenvatinib in the treatment of patients with ATC, a disease with a poor prognosis. Despite the use of aggressive therapy, median survival has not changed significantly in several decades (15). Current survival times for patients with ATC are reported to be extremely short and median OS for patients with unresectable disease ranges from 0.5 to 6 months despite multimodal treatment (16–18).

The key finding of this study is that the median OS (~8 months) using our treatment regimen was improved compared with historical OS rates for currently available therapies. Furthermore, tumor volume decreased by ≥50% in 33% of patients, and showed at least some reduction in 88.9% of the patients receiving lenvatinib. These results, in patients who had progressed on their existing treatment, suggest that the addition of lenvatinib can provide benefit even to patients with advanced disease who otherwise have few treatment options.

In recent years, the focus of cancer research has turned to molecular targeted therapies, and in ATC the most promising new treatment options appear to be multitargeted TKIs (1, 19, 20). However, although early stage studies have reported encouraging results, phase 3 data are lacking, and the place of these treatment agents within the therapeutic algorithm remains unclear. Retrospective data can be used to bridge the current knowledge gap, and provide much-needed data for clinicians looking to improve outcomes for their patients. Previous retrospective analyses in Japanese patients have indicated that lenvatinib is associated with disease control rates (clinical response or stable disease) of 44% (21) to 100% (22), and OS times of 165 (22) to 166 (21) days, although tumor volumes were not evaluated. While it is not possible to directly compare studies due to the heterogeneity of the enrolled patients and the differing therapeutic procedures used, the treatment regimen used in our institute was able to produce a relatively lengthy median OS of 230 days, and additionally demonstrated a direct, measurable impact on tumor volume in almost 90% of patients.

A recent phase 2 study of lenvatinib treatment in patients with ATC reported a median OS of 10.6 months and some degree of tumor shrinkage (10, 14). The results of our analysis support these clinical study data and confirm the hypothesis that lenvatinib should be considered earlier in the treatment regimen. Currently, there is no established protocol for the use of lenvatinib in patients with ATC. Our data suggest that the addition of lenvatinib to existing anticancer drugs may increase the sensitivity to treatment; this confirms previous non-clinical research which suggested synergistic antineoplastic activity resulting from the combined use of chemotherapy, radiotherapy, and TKI inhibitors (23). However, the potential for toxicity may also increase when combining several treatment modalities; furthermore, AEs are commonly reported with TKI treatment (24, 25). Nonetheless, these may be overcome through dose adjustments (24, 25), and the benefit accorded to patients through the early initiation of lenvatinib in combination with other anticancer therapies may outweigh the potential risks.

### Study Strengths and Limitations

This article presents important and novel data on the use of lenvatinib to treat ATC, which have not been available thus far. There is a clear unmet need for improved treatment regimens for patients with ATC, who currently have a poor prognosis and short survival times, and this analysis expands the evidence base for clinicians considering the optimal therapy for ATC. We recognize that in our study, patients were only treated with lenvatinib following progression on paclitaxel and IMRT. Although we hypothesize that introducing lenvatinib into the treatment regimen at an earlier stage may help to increase survival times, the burden on the patient must also be considered. As such, optimal adjustment of the appropriate doses of lenvatinib and paclitaxel will be critical.

We also acknowledge the study limitations of our analysis, several of which are inherent to retrospective analyses. First, this was an observational study, and the results were reliant on the completeness and accuracy of the medical records used. In addition, the lack of a control arm of patients who did not receive lenvatinib treatment precludes direct comparisons; nonetheless, historical comparisons with previously published studies suggest the utility of lenvatinib in this setting. Second, data from this single-center study require additional confirmation before they can be extrapolated to the wider ATC population. Moreover, as this study only included Korean patients, the generalizability of the current findings may be limited. Finally, there was no attempt made in this study to evaluate the type and frequency of AEs related to lenvatinib therapy. ATC is a very aggressive disease that causes multiple symptoms in patients. In addition, AEs may be caused by paclitaxel or IMRT treatment. Because this was a retrospective analysis of electronic medical records, we were not able to investigate the association, if any, between AEs and lenvatinib. Additional analyses will be necessary to further elucidate the safety profile of this treatment regimen.

### Conclusions

The results of this analysis of patients with ATC who had progressed on prior therapy suggest that the addition of lenvatinib was able to improve survival duration and reduce tumor volume. Further studies to evaluate lenvatinib in this patient population are warranted.

## Data Availability Statement

The raw data supporting the conclusions of this article will be made available by the authors, without undue reservation.

## Ethics Statement

Ethical review and approval was not required for the study on human participants in accordance with the local legislation and institutional requirements. Written informed consent for participation was not required for this study in accordance with the national legislation and the institutional requirements.

## Author Contributions

SK, S-MK, HC, H-SC, and CP contributed to the conception and design of the study. SK, HC, B-WK, and YL organized the database. SK and S-MK performed the statistical analysis. SK and S-MK wrote the first draft of the manuscript. SK and S-MK wrote sections of the manuscript. All authors contributed to manuscript revision, read, and approved the submitted version.

## Conflict of Interest

The authors declare that the research was conducted in the absence of any commercial or financial relationships that could be construed as a potential conflict of interest.
