# Supplementary material for: Survival With Lenvatinib for the Treatment of Progressive Anaplastic Thyroid Cancer: A Single-Center, Retrospective Analysis
Source: Front Endocrinol (Lausanne). 2020 Sep 2;11:599. doi: 10.3389/fendo.2020.00599 (PMC7492269; doi:10.3389/fendo.2020.00599)
Supplement: Supplementary file 1 [file Data_Sheet_1.docx]

Supplementary Material

## *Table S1. Individual patient data*

| **Patient** | **Sex** | **Age** | **Tumor size**  **(cm)** | **Staging** | **Operation** | **Chemotherapy  cycles** | **Radiation**  **doses** | **Lenvatinib  use (days)** | **Survival status** | **Survival (days)** |
| --- | --- | --- | --- | --- | --- | --- | --- | --- | --- | --- |
| 1 | F | 74 | 4.8 | 4b | No | 1 | 3360 | 363 | Deceased | 402 |
| 2 | F | 52 | 3.1 | 4c | R2 | 6 | 5060 | 65 | Deceased | 343 |
| 3 | F | 57 | 5 | 4c | R1 | 6 | 6300 | 228 | Deceased | 296 |
| 4 | M | 60 | 5.5 | 4c | R1 | 5 | 6000 | 367 | Deceased | 839 |
| 5 | M | 72 | 5.7 | 4c | No | 6 | 6300 | 175 | Deceased | 224 |
| 6 | F | 62 | 6.1 | 4c | No | 3 | 5250 | 55 | Deceased | 105 |
| 7 | F | 68 | 4.3 | 4c | No | 5 | 5880 | 147 | Deceased | 230 |
| 8 | F | 75 | 5.8 | 4c | No | 1 | 4830 | 31 | Deceased | 64 |
| 9 | M | 56 | 2.3 | 4c | R2 | 5 | 6600 | 189 | Deceased | 356 |
| 10 | F | 64 | 3.5 | 4c | No | 1 | 3080 | 333 | Living | 390 |
| 11 | F | 70 | 6.5 | 4c | No | 5 | 4400 | 118 | Deceased | 230 |
| 12 | F | 61 | 6.8 | 4c | No | 4 | 5500 | 41 | Deceased | 131 |
| 13 | F | 42 | 6.8 | 4c | R2 | 3 | 3960 | 107 | Deceased | 152 |
| 14 | M | 70 | 8.9 | 4c | No | 1 | 2200 | 65 | Deceased | 131 |
| 15 | F | 59 | 4.7 | 4c | No | 2 | 6600 | 133 | Deceased | 183 |
| 16 | M | 84 | 3.4 | 4c | No | 1 | 2420 | 181 | Living | 279 |
| 17 | M | 56 | 4.4 | 4c | R2 | 1 | 6000 | 243 | Living | 265 |
| 18 | M | 86 | 5.6 | 4c | No | 3 | 6600 | 26 | Deceased | 78 |

## *Table S2. Treatment schedule*

| Week | Sun | Mon | Tue | Wed | Thu | Fri | Sat |
| --- | --- | --- | --- | --- | --- | --- | --- |
| 0 |  |  |  | Pre-treatment PET |  |  |  |
| 1 |  | Day 1  1-1 chemotherapy + radiation start |  |  |  |  | Day 7 |
| 2 |  | Day 8  1-2 chemotherapy |  |  |  |  | Day 14 |
| 3 |  | Day 15  1-3 chemotherapy |  |  |  |  | Day 21 |
| 4 |  |  |  | 18 fractions  PET-CT limited – operation decision |  |  | Day 28 |
| 5 |  | Day 29  2-1 chemotherapy |  |  |  | Operable - 25 fractions  radiation finish | Day 35 |
| 6 |  | Day 36  2-2 chemotherapy |  |  |  | 30 fractions  radiation finish | Day 42 |
| 7 |  | Day 43  2-3 chemotherapy |  |  |  |  | Day 49 |
| 8 |  | Neck CT  Chest CT |  |  |  | Operation | Day 56 |
| 9 |  | Day 64  3-1 chemotherapy |  |  |  |  | Day 63 |
| 10 |  | Day 71  3-2 chemotherapy |  |  |  |  |  |
| 11 |  | Day 78  3-3 chemotherapy |  |  |  |  |  |
| 14 |  |  |  | PET-CT follow-up |  |  |  |

CT, computed tomography; PET, positron emission tomography.
